# Neovascular prostate specific membrane antigen (PSMA) expression in bone and soft tissue sarcoma: a systematic analysis

**DOI:** 10.1007/s00428-025-04086-6

**Published:** 2025-04-09

**Authors:** Irene A. Spiridon, Sheena L. M. Ong, Jiri Soukup, Oana-Maria Topirceanu-Andreoiu, Lioe-Fee de Geus-Oei, Hans Gelderblom, Suk Wai Lam, Inge H. Briaire de Bruijn, Brendy E. W. M. van den Akker, Linda Hijmen, Karoly Szuhai, Judith V. M. G. Bovée

**Affiliations:** 1 Department of Pathology, Leiden University Medical Center, Postzone L1-Q, Postbus 9600, 2300 RC Leiden, The Netherlands; 2 Department of Pathology, “Grigore T. Popa” University of Medicine and Pharmacy Iasi, Iasi, Romania; 3 Department of Pathology, Military University Hospital Prague, Prague, Czech Republic; 4 The Fingerland Department of Pathology, Charles University, Faculty of Medicine in Hradec Králové and University Hospital Hradec Králové, Sokolska 581, 500 05 Hradec Kralove, Czech Republic; 5 Department of Pathology, Marie Curie Children’s Clinical Hospital & OncoTeam Diagnostic, Bucharest, Romania; 6 Department of Radiology, Leiden University Medical Center, Leiden, The Netherlands; 7 Department of Medical Oncology, Leiden University Medical Center, Leiden, The Netherlands; 8 Department of Nuclear Medicine, Leiden University Medical Center, Leiden, The Netherlands; 9 Department of Cell and Chemical Biology, Leiden University Medical Center, Leiden, The Netherlands

**Keywords:** PSMA, Immunohistochemistry, Soft tissue sarcoma, Bone tumors, Osteosarcoma, Giant cell tumor of bone, Angiosarcoma, Hemangioma

## Abstract

Bone and soft tissue sarcomas are a highly heterogeneous group of rare cancers of mesenchymal origin. Treatment options other than surgery have limited efficacy due to low response rates with some exceptions. Radioligand therapy targeting prostate-specific membrane antigen (PSMA) may provide a novel treatment option, as it was recently suggested that soft tissue sarcomas express PSMA in the neovasculature, and on PET/CT imaging, multiple sarcomas have shown intense PSMA-tracer accumulation. Moreover, in prostate cancer patients, incidental PSMA uptake was seen in hemangiomas. In addition to confirming previous results in soft tissue sarcoma, the current study aims to systematically explore the expression of PSMA in bone tumors and in vascular tumors. Immunohistochemistry for PSMA was performed on a total of 706 tumors. High PSMA expression in the neovasculature was seen in 29% of the soft tissue sarcomas and 33% of the bone sarcomas. Malignant tumors showed a higher frequency of PSMA expression (score 2) as compared to benign tumors, with a high frequency in rhabdomyosarcoma (2 of 2, 100%), mesenchymal chondrosarcoma (14 of 20, 70%), undifferentiated sarcoma of bone (4 of 6, 67%) and of soft tissue (13 of 20, 65%), and osteosarcoma (46 of 81, 57%). In addition, giant cell tumor of bone displayed a high PSMA labelling in 67% of the cases. In contrast, high PSMA expression was seen in only 0–40% of the non-neoplastic vessels in vascular tumors, while 8% of them expressed PSMA in the tumor cells. Thus, with variable frequency among the different subtypes, a subset of bone and soft tissue tumors, both malignant and intermediate behavior, express PSMA and these patients may benefit from PSMA-targeting PET/CT scans or PSMA targeted radioligand therapy.

**Supplementary Information:**

The online version contains supplementary material available at 10.1007/s00428-025-04086-6.

## Introduction

Sarcomas are malignant mesenchymal tumors with heterogenous anatomical distribution, that arise in bone, fat, cartilage, muscle, nerves, blood vessels, or other connective tissue. While they are less frequent than tumors of epithelial origin (i.e., < 6 cases/100,000 individuals), they represent a complex challenge in terms of biological behavior and response to standard therapy [1]. With more than 70 soft tissue and bone sarcoma subtypes described, their histological complexity and molecular heterogeneity requires constant updating of current classification systems [2]. Management of patients is challenging, as early diagnosed cases harbor a good prognosis following radical resection, while cases with metastatic presentation are most often unresectable and show resistance to standard chemotherapy regimens [3].

Progressive understanding of the complex pathogenesis of sarcomas and their acquisition of treatment-resistance mechanisms via “omics” sciences has paved the way for tailoring treatment to the particularities of each patient [4], with an explosion of novel research in the field of immunotherapy [5]. In parallel, genetic alterations that generate the upregulation of various molecules in cancer cells have been the subject of theranostics, which use radiolabeled compounds to effectively target advanced tumors with relatively low systemic side effects [6]. Recent publications in the field of sarcoma highlight the potential of molecules such as fibroblast activation protein alpha (FAPα) present in the tumor microenvironment (TME) [7] or prostate-specific membrane antigen (PSMA) expressed by the neovasculature of the tumor [8] as promising targets for the radiotherapy of these malignancies [9].

PSMA is a well-characterized glycoprotein first discovered in prostate tissue [10]. It is part of the type II transmembrane glycoproteins family and encode by the *FOLH1* (folate hydrolase 1) gene [11]. However, other normal cells such as glial or epithelial duodenal cells also show expression of this protein, its function being site-dependent and linked to glutamate synthesis or breakdown [12]. PSMA is overexpressed in prostate carcinoma, where it plays a key role in promoting carcinogenesis through release of glutamate that leads to activation of the PI3K/AKT pathway [13]. Its expression has been reported to be confined to the tumor-associated neovasculature of a wide variety of other cancers, including soft tissue and bone sarcomas [10, 14–16]. Some authors highlight the link between tissue protein expression and the outcome of patients suggesting its use as a prognostic biomarker, as is the case of ovarian cancer [16]. In the field of theranostics, the use of PSMA for imaging in patients with advanced prostate cancer has led to the incidental detection of extra-prostatic concurrent tumors showing increased uptake of the radiolabeled compound, including sarcomas [17, 18]. Therefore, PSMA-targeting PET/CT scans and eventually PSMA-targeted radioligand therapy may be of benefit for patients with soft tissue and bone sarcomas that express PSMA.

Heikötter et al. systematically analyzed PSMA expression in soft tissue tumors and Ewing sarcoma, and showed that PSMA-expression was more prevalent in malignant than benign mesenchymal tumors. Immunohistochemical PSMA expression varied between 0 and 86% among different histological subtypes, showing expression in about one third of patients and strong expression in more than 10% of cases. So far, a systematic analysis across bone sarcomas is lacking, with only one paper previously reporting expression in osteosarcomas [19].

PSMA expression is mainly confined to the neovasculature of tumors and incidental findings of PSMA uptake were observed in hemangiomas in prostate cancer patients, suggesting overexpression of PSMA in vascular tumors [19–21]. Furthermore, PSMA has been shown to regulate angiogenesis through integrin signal transduction [11]. Thus, we set out to perform a systematic characterization on the tissue expression of PSMA in a broad array of soft tissue and bone tumors with a special focus on bone sarcoma and vascular tumors.

## Material and methods

### Sample collection

Tissue microarrays of previously published cohorts [22, 23] were used to evaluate the immunohistochemical expression of PSMA. In general, all TMAs contained cores of 2 mm in diameter, with the exception of the angiosarcoma TMA (0.6 mm). For every tumor, if possible, three cores were present in the TMA block. The cohort of bone tumors included osteochondroma (*n* = 3), enchondroma (*n* = 3), conventional chondrosarcoma (*n* = 50), dedifferentiated chondrosarcoma (*n* = 28), clear cell chondrosarcoma (*n* = 16), mesenchymal chondrosarcoma of bone (n = 10), undifferentiated spindle cell sarcoma (*n* = 6), osteosarcoma (*n* = 86 including telangiectatic osteosarcoma (*n* = 5)), aneurysmal bone cyst (*n* = 6), chordoma (*n* = 85), giant cell tumor of bone (*n* = 66), angiosarcoma of bone (*n* = 24), hemangioma (*n* = 7), and epithelioid hemangioma (*n* = 8). The soft tissue tumors cohort included leiomyosarcoma (*n* = 43), uterine leiomyoma (*n* = 7), undifferentiated pleomorphic sarcoma (*n* = 20), malignant peripheral nerve sheath tumor (*n* = 35), schwannoma (*n* = 20), neurofibroma (*n* = 11), myxofibrosarcoma (*n* = 17), radiation‐associated sarcoma (*n* = 8, including two cases of osteosarcoma, two cases of angiosarcoma, and four cases of undifferentiated spindle cell sarcoma), rhabdomyosarcoma (n = 2), synovial sarcoma (n = 61), pleomorphic liposarcoma (n = 4), mesenchymal chondrosarcoma of soft tissue (*n* = 10), angiosarcoma (*n* = 44), epithelioid hemangioendothelioma (*n* = 5), pseudomyogenic hemangioendothelioma (*n* = 1), hemangioma (*n* = 16), and vascular malformations (*n* = 2). Survival- and disease progression–related data were only available for the conventional chondrosarcoma group.

### Immunohistochemistry

Immunohistochemistry was performed with a commercially available antibody (Cell Signaling Technology anti-PSMA rabbit monoclonal antibody, clone D7I8E, dilution 1:50) using a standard laboratory protocol, as described previously [23]. To summarize, antigen retrieval by a microwave method was performed on deparaffinized sections in Citrate (pH 6.0). This step was followed by overnight incubation with the primary antibody. Subsequent detection and visualization of DAB + substrate chromogen (Dako, Glostrup, Denmark) was done using power vision poly‐HRP (ImmunoLogic, the Netherlands). As a final step, all the slides were counterstained with hematoxylin, dehydrated and mounted.

### PSMA expression evaluation

PSMA scoring in tumor neovasculature was assessed independently by two pathologists using the previously established method described in detail by Heitkotter et al. [24]. In brief, staining intensity was scored as negative (0), weak (1 = barely perceptible staining at high power (400 ×) magnification), moderate (2 = readily apparent at low power (40 ×) magnification) or strong (3). The semiquantitative expression threshold of PSMA positive cells was set at 5%. The low expression group (PSMA labelling index = 1) included weak (1) or moderate (2) staining intensity in < 5% of the neovasculature and a weak (1) staining intensity in > 5% of the neovasculature. To account for heterogeneous expression in different cores of the same tumor, a global overall score per case was established by performing a cumulative evaluation of the tumor surface area in the cores evaluating the predominant pattern. Three groups were defined. The negative expression group included cases with no identifiable PSMA immunoreactivity in neovasculature or tumor cells. The strong expression group (PSMA labelling index = 2) included the cases with moderate (2) staining intensity in > 5% of the neovasculature and a strong (3) staining intensity regardless of the fraction of stained cells. The presence of PSMA staining in the tumor cells was noted separately. Discrepant cores were jointly re-evaluated to reach a consensus score. When a consensus could not be reached, the case was eliminated from the cohort.

### Statistical analysis

Statistical analysis was performed using SPSS v.27 software (SPSS Inc., Chicago, USA). Statistical analyses between the two groups were performed using *t*-test, *χ*^2^ test, or Fisher’s exact test. Disease-free survival was defined as the interval between the date of histopathological diagnosis and the date of the first detection of recurrence or the date of the last known follow-up without evidence of recurrence. Overall survival time was censored at the time of the last visit for regular follow-ups. Survival rates were estimated using the Kaplan–Meier method and compared by the log-rank test. Statistical results were considered significant if *p* < 0.05.

## Results

### PSMA expression observed in different subsets of soft tissue and bone sarcoma

In normal tissues, PSMA was expressed in the epithelial lining of the small bowel, in hepatocytes and in sweat glands (Suppl Fig. 1). A total of 704 tumors were analyzed, including 78 benign tumors, 77 tumors of uncertain malignant potential, and 549 malignant tumors. The majority (74.4%) of benign tumors did not express PSMA, with only 7.7% categorized as PSMA labelling index 2. Among the malignant tumors, the cases were almost evenly distributed in the three categories of PSMA expression. Expression of PSMA was confined to the neovasculature and was absent in tumor cells. Intermediate malignant potential tumors displayed a great proportion of cases with high PSMA expression (59.7%), which was mainly caused by the relatively high frequency in giant cell tumor of bone (66.7%) (Table 1). PSMA index was correlated with biological category (*p* < 0.00001).

**Table 1:** PSMA expression in 704 soft tissue and bone tumors according to biological behavior

*Biological behavior*	*PSMA index 0*	*Negative for PSMA*	*PSMA index 1*	*Low PSMA expression*	*PSMA index 2*	*Strong PSMA expression*	*Total*
*Benign*	**58**	***74.36%***	**14**	***17.95%***	**6**	***7.69%***	78
· ***Soft tissue tumors***	43	***84.31%***	7	***13.73%***	1	***1.96%***	51
· ***Bone tumors***	15	***55.56%***	7	***25.93%***	5	***18.52%***	27
*Intermediate*	**18**	***23.38%***	**13**	***16.88%***	**46**	***59.74%***	77
· ***Soft tissue tumors***	7	***63.64%***	2	***18.18%***	2	***18.18%***	11
· ***Bone tumors***	11	***16.67%***	11	***16.67%***	44	***66.67%***	66
*Malignant*	**212**	***38.62%***	**164**	***29.87%***	**173**	***31.51%***	549
· ***Soft tissue tumors***	106	***43.44%***	67	***27.46%***	71	***29.10%***	244
· ***Bone tumors***	106	***34.75%***	97	***31.80%***	102	***33.44%***	305
***Total***	***288***	***40.91%***	***191***	***27.13%***	***225***	***31.96%***	***704***

**Fig. 1:**
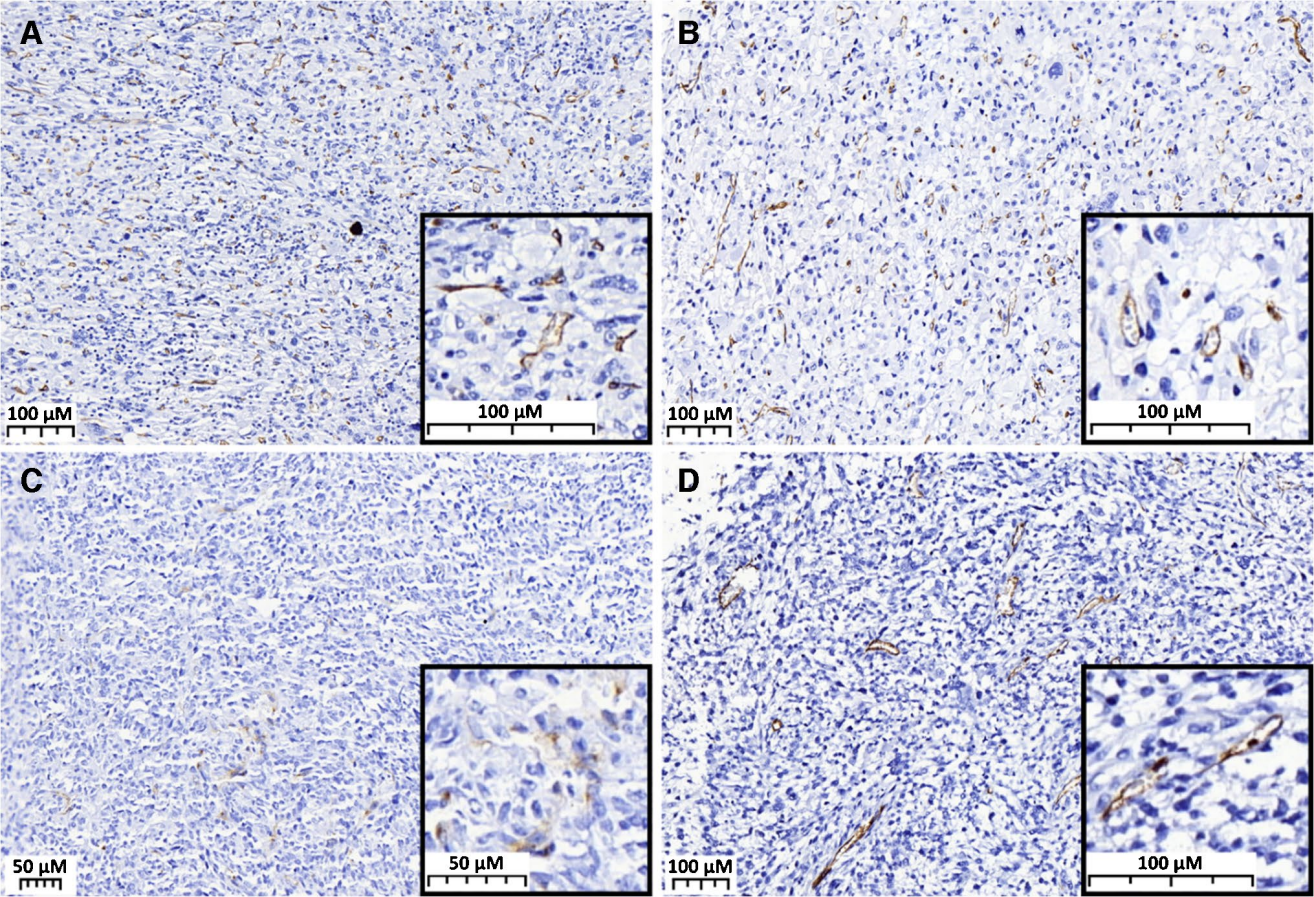
PSMA expression in neovasculature of soft tissue sarcomas. **A** MPNST showing intense and diffuse expression of PSMA. **B** Undifferentiated pleomorphic sarcoma with PSMA index 2. **C** Synovial sarcoma with PSMA index 1. **D** Leiomyosarcoma with diffuse and intense expression of PSMA in the neovasculature

**Fig. 2:**
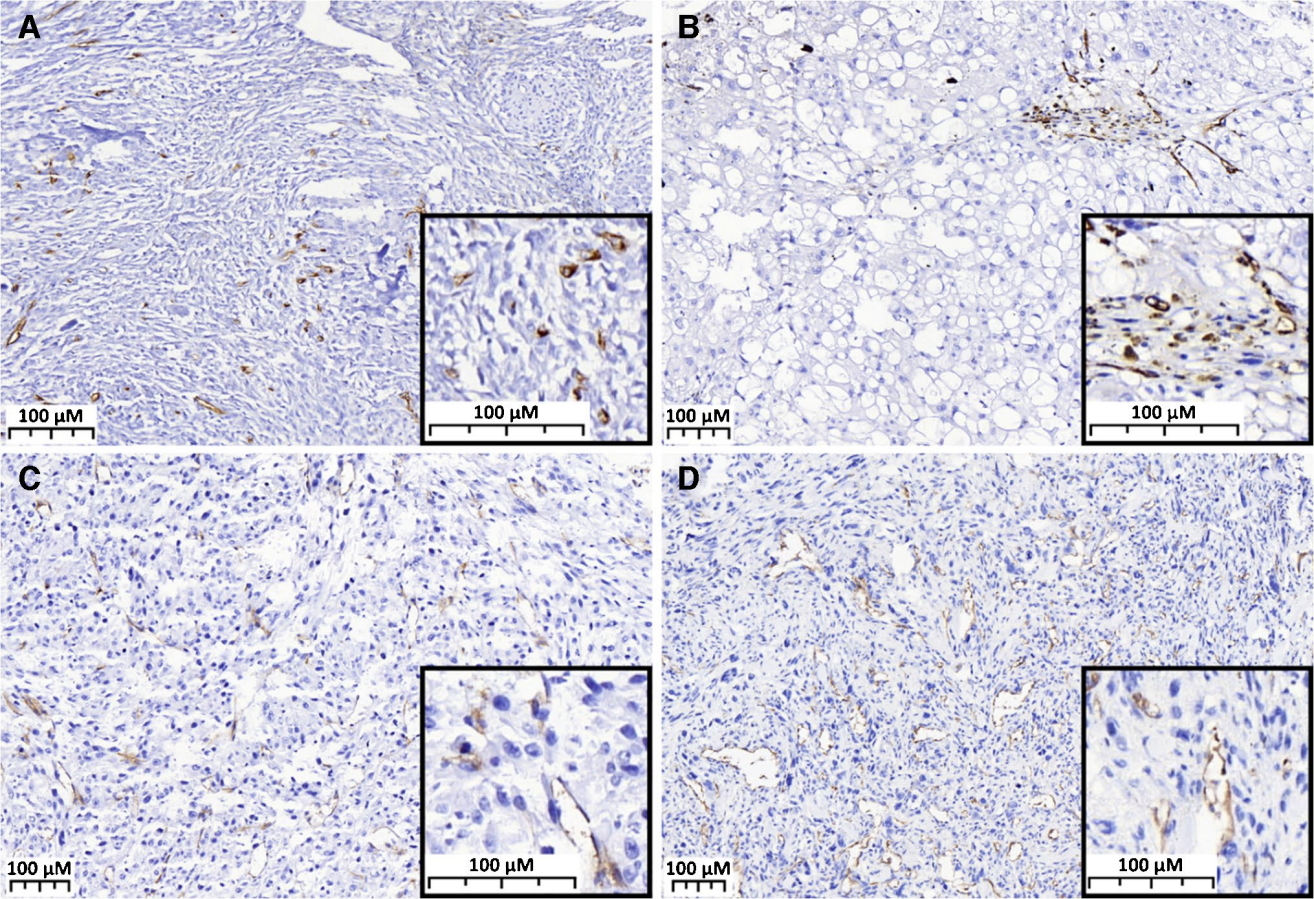
PSMA expression in the neovasculature of bone tumors. **A** ABC showing intense and diffuse expression of PSMA. **B** Chordoma showing intense and diffuse expression of PSMA. **C** Dedifferentiated chondrosarcoma with PSMA index 1 in the dedifferentiated component. **D** Osteosarcoma with PSMA index 2

PSMA expression was found to be confined to the neovasculature in sarcoma, and the frequency of positivity, defined as PSMA labelling index 1 (“low expression group”) and 2 (“strong expression group”), was highly variable among sarcoma subtypes (Table 1). On average, 24.1% of soft tissue tumors (Fig. 1) and 37.9% of bone tumors (Fig. 2) displayed strong labelling for PSMA (index 2) (Table 2).

**Table 2:**
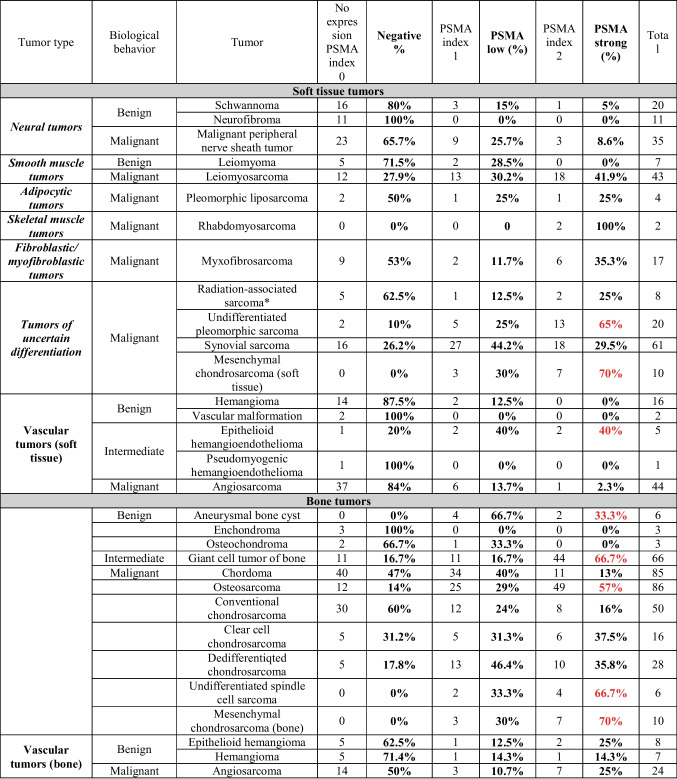
PSMA expression in soft tissue and bone tumors according to malignant potential and subtypes

Strongest PSMA index 2 staining is colored in red

*Radiation induced sarcoma group included: two cases of osteosarcoma, two cases of angiosarcoma, and four cases of undifferentiated spindle cell sarcoma

**Fig. 3:**
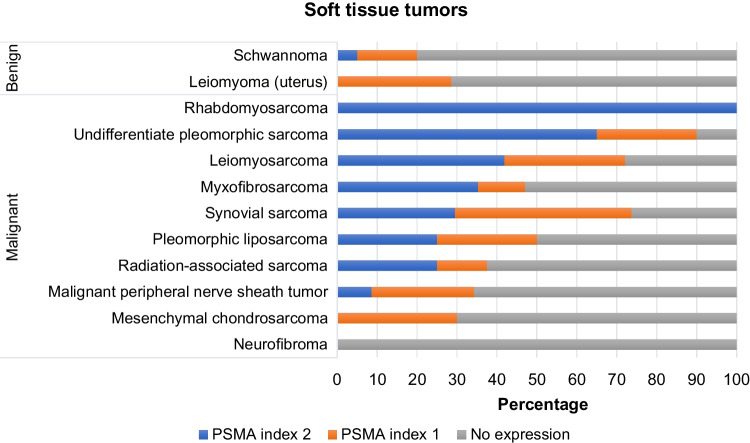
Distribution of PSMA expression in soft tissue tumors

The highest frequency of positivity (labelling index 1 or 2) among the soft tissue sarcomas was seen in mesenchymal chondrosarcoma (100%), undifferentiated pleomorphic sarcoma (90%), and leiomyosarcoma (72.1%) (Fig. 3). A labelling index of 2 was observed in 41.8% of leiomyosarcomas, 35.3% of myxofibrosarcomas, and 70% of mesenchymal chondrosarcomas of soft tissue. On the other hand, the presence of PSMA expression was relatively low in MPNST (34.2%), with only 8.6% of cases showing strong expression.

**Fig. 4:**
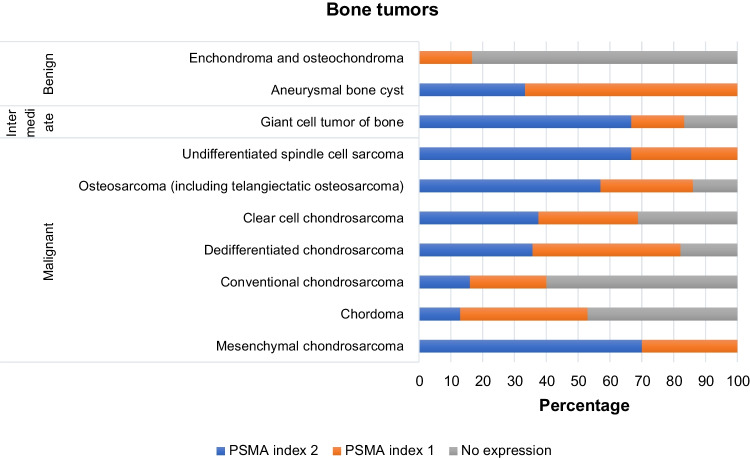
Distribution of PSMA expression in bone tumors

Despite the limited number of cases, PSMA staining was strong and diffuse in the neovasculature of both rhabdomyosarcomas that were included (two cases, 100%). Of interest, among the high-grade bone sarcomas, 81.9% of conventional high-grade osteosarcomas, including four out of five teleangiectatic osteosarcomas, showed positive PSMA expression (labelling index 1 or 2) of the neovasculature (Fig. 4). Among the positive cases, over half of the tumors showed strong expression for PSMA (57%).

Conventional chondrosarcomas were predominantly negative for PSMA expression (PSMA score 0–60%), with only a small percentage of tumors showing high levels of PSMA (score 2–16%). Expression of PSMA did not correlate with histological grade (*p* = 0.3916), nor with overall survival (*p* = 0.1258) or disease-free survival (*p* = 0.1324). Among the chondrosarcoma subtypes, 70% of mesenchymal chondrosarcomas, considered a high-grade bone sarcoma, showed strong PSMA expression in the neovasculature, while clear cell chondrosarcoma, considered to be of low to intermediate grade, showed strong PSMA labelling in 37.5% of the tumors. In the case of dedifferentiated chondrosarcoma, PSMA expression was evaluated exclusively in the dedifferentiated component and proved strong (labelling index 2) in approximately one third of cases (PSMA score 2–35.7%).

Among the benign and intermediate lesions, enchondroma and osteochondroma showed a predominantly absent expression of PSMA (100% and 66.7%, respectively), while giant cell tumors of bone were most often positive, showing strong labelling (index 2) in 66.6% of cases. Of note, also all aneurysmal bone cysts in the cohort were positive, though the predominant staining pattern was with PSMA labelling index of 1 (66.6%).

### PSMA is not over-expressed in vascular tumors

**Fig. 5:**
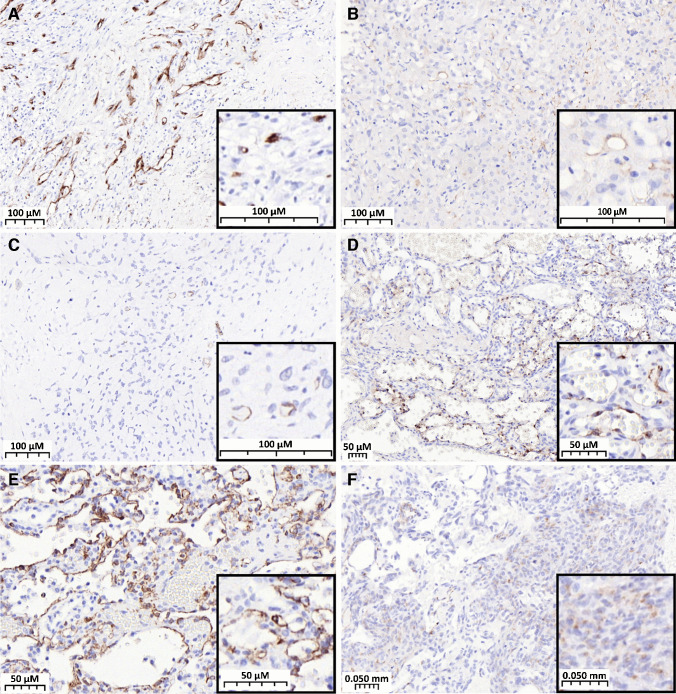
PSMA expression in vascular tumors. **A** Angiosarcoma of bone showing intense and diffuse expression of PSMA in the neovasculature (PSMA score 2), the tumor cells are negative. **B** Epithelioid hemangioma with PSMA index 1. **C** Epithelioid hemangioendothelioma with PSMA index 1. **D** hemangioma of the liver showing expression of PSMA in the endothelial cells forming the tumor (PSMA index 2). **E** Strong PSMA expression in the neoplastic endothelial cells in hemangioma of bone. **F** Weak expression (PSMA index 1) in the tumor cells of angiosarcoma of soft tissue

**Fig. 6:**
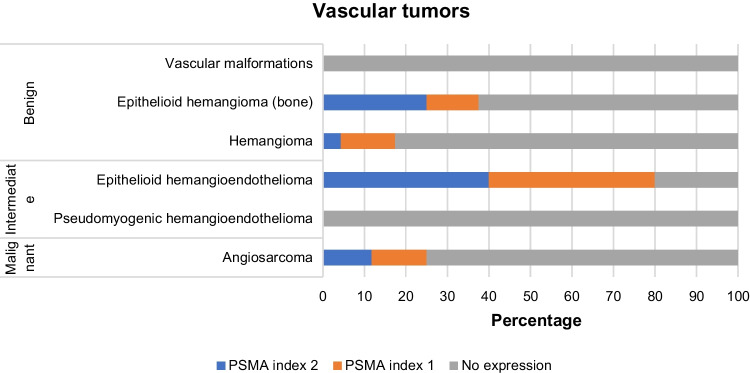
Distribution of PSMA expression in the neovasculature of vascular tumors

Since PSMA expression is confined to the tumor neovasculature, and several case reports demonstrated high PSMA uptake in vascular tumors [20, 21], we extended our cohort to include benign, intermediate, and malignant vascular tumors of bone and soft tissue (Fig. 5). Benign vascular tumors such as hemangioma are predominantly negative for PSMA (77.4%), showing little expression in the neovasculature, with only four cases (12.9%) of score 1 and three cases (9.67%) of score 2. From the total of five cases of epithelioid hemangioendothelioma (EHE), four (80%) were positive for PSMA and strong staining (labelling index 2) of the neovasculature was observed in 40% of cases. The positivity rate among angiosarcomas of soft tissue was only 15.9% and 35.7% in the case of tumors with bone localization, with a PSMA labelling index of 2 observed mainly in tumors located in the bone (25% of bone angiosarcomas) (Fig. 6) (Table 2).

PSMA expression was occasionally also observed in the tumor cells, as follows. Low PSMA expression (score 1) was noted in five out of 68 angiosarcoma (7.35%) and two out of 31 cases of hemangioma (6.45%). Strong expression (score 2) was found in two cases of hemangioma (6.45%), with bone and liver localization. Thus, expression of PSMA in tumor cells of vascular neoplastic proliferations was rare, accounting for 8% of the vascular tumors (nine out of 107 cases). Only the staining at the level of the neovasculature was taken into account for quantification.

### Intratumoral heterogeneity of PSMA expression

Since we used tissue microarrays to evaluate positivity of the neovasculature, we observed discrepancies between the different cores belonging to the same case, meaning different scores were attributed to the cores belonging to the same case (Table 2). This was defined a heterogenous pattern of intratumoral PSMA expression and was identified in a little over one third of the tumors in our cohort (28.1%). Of note, the highest rate of intratumoral heterogeneity in terms of PSMA expression was observed in mesenchymal and dedifferentiated chondrosarcoma, of which half of the tumors (50% of cases) displayed variable staining between cores, where only 12.5% of conventional chondrosarcomas showed heterogeneity. In osteosarcoma, the neovasculature showed variable levels of PSMA staining in 30.8% of cases, while the rate of heterogeneity was highest for synovial sarcoma (40.9%).

## Discussion

PSMA expression in the tumor-associated neovasculature of sarcomas is a relatively novel topic that is strongly linked to the development of theranostics. The literature includes a limited number of isolated case reports, where PSMA uptake was observed in soft tissue sarcoma [17, 25–28] and a positive response of leiomyosarcoma to 117Lu-PSMA radioligand therapy was reported [29]. A systematic analysis of PSMA expression in the soft tissue sarcoma neovasculature was reported by Heitkotter et al. [24]. In the present study, the aim was to confirm and supplement their findings by focusing predominantly on bone sarcomas and vascular tumors. Soft tissue and bone sarcomas that express PSMA could potentially benefit from PSMA-targeted radioligand therapy.

In the articles by Heitkötter et al., over 700 soft tissue and bone tumors of various subtypes were analyzed, with an overall high percentage of PSMA positivity (scores 1 and 2) of 19.38% of cases at the level of the tumor neovasculature [24]. The current paper reveals an overall higher frequency of PSMA expression (scores 1 and 2) (59%) in the current cohort of 704 tumors covering the entire spectrum of biological behavior from benign to malignant tumors of soft tissue and bone, including vascular tumors (Table 1). In terms of tumor biological behavior, a notable difference between the two studies is the percentage of intermediate tumors harboring a strong PSMA expression (59.7% in our study vs. 2.03%), owing predominantly to the inclusion of giant cell tumor of bone with a frequency of 66.7% in our cohort [24]. The study of Heitkötter et al. highlights strong positivity for PSMA especially in malignant tumors (6.51%), a value which was exceeded in our study (31.5%), owing to both malignant soft tissue tumors (29.1%) and malignant bone tumors (33.4%) (Table 1).

For soft tissue tumors, a high percentage of synovial sarcomas (29.5%) showed strong expression of PSMA, which is in accordance with previously reported findings [24]. In our study, the rate of cases with PSMA labelling index 2 was higher than in the previous report for pleomorphic liposarcoma (25% vs. 10%), undifferentiated soft tissue sarcoma (65% vs. 18.2%). and myxofibrosarcoma (35% vs. 0%). MPNST showed a PSMA positivity in one third of cases (34.2%), with only a minority classified as having strong expression (8.5%). Our data is in accordance with the study of Heitkötter et al. [24] and in in contradiction with a previous report that describes complete negativity of these tumors for PSMA [30].

The current study predominantly focused on bone tumors, as they often pose a therapeutic challenge, particularly for locally advanced sarcomas. We did not include Ewing sarcoma, as that was the only bone sarcoma represented in the study of Heitkötter et al. [24], showing a PSMA labelling index of 1 in six out of 106 cases, while none of them showed strong expression. For high-grade conventional osteosarcoma, the most frequent bone sarcoma, the data available in the literature is limited to one study, with a cohort of 45 cases showing positivity for PSMA in the neovasculature in 47% of tumors [19]. The current study shows that 86% of osteosarcomas display some level of PSMA expression and 56.9% show a strong expression pattern. It is crucial to note that in the studies available in the literature PSMA staining in sarcoma has been conducted using several commercially available antibodies and that only recently a system of quantification of protein tissue expression has been developed [24].

Of particular interest, our study uncovered strong positivity for PSMA in 67% of giant cell tumor of bone from the total of 66 cases analyzed. Giant cell tumor of bone demonstrates intermediate biological behavior (locally aggressive, rarely metastasizing) and can be challenging in the context of local recurrence or particular anatomical presentation which can require extensive surgical interventions. Our data is confirmed by a recent paper evaluating the expression of PSMA on FFPE tissue samples of 28 patients, showing that 71.4% of tumors were positive for PSMA (albeit not applying the same scoring method as described by Heitkötter et al. and that this expression was restricted to the neovasculature [24]. Furthermore, the authors argue the important role of PSMA-targeted radiopharmaceuticals in the management of giant cell tumor of bone by demonstrating PSMA-specific fluorescent probe (FAM-C6-1298) uptake in PSMA positive cells, indicating successful targeting of these tumors in vitro [31].

In our cohort, conventional chondrosarcoma expressed PSMA in over one third of cases. However, a PSMA index of 2 was more frequently observed in the more aggressive subtypes such as dedifferentiated chondrosarcoma (35.8%). Of note, mesenchymal chondrosarcoma displays a strong PSMA expression pattern (70%) regardless of primary localization in bone or soft tissue. In contrast to previous studies, where PSMA expression was associated with histological grade and outcome, for conventional chondrosarcoma such a correlation was not found.

The incidental findings of PSMA uptake in hemangioma in patients with prostate cancer and the involvement of PSMA in regulating angiogenesis, led us to investigate the expression of PSMA in different subtypes of benign, intermediate, and malignant vascular tumors, where we expected PSMA expression in the neoplastic tumor cells. This was however very rare (8%), which is in line with the low frequency of PSMA uptake in hemangiomas seen clinically. PSMA expression was demonstrated in the neovasculature in only few EHE and angiosarcoma cases. Vascular tumors of intermediate malignancy, which show locally aggressive features such as epithelioid hemangioendothelioma, were most frequently positive (80%), and a PSMA index of 2 was identified in 40% of these tumors. Moreover, our study revealed that a subset of angiosarcomas of soft tissue (15.9%) are positive for PSMA (score 1 and score 2), findings which are in line with the previous report [24], while primary angiosarcoma of bone showed PSMA positivity in a higher number of cases (35.7%).

The use of tissue microarrays, preferred for feasibility and representative of the real-world situation where expression would need to be assessed on small needle biopsies, is a limitation of the study as we showed that expression may be heterogeneous and may vary between the tissue cores of the same patient within the microarray: 28.1% of the cases in our cohort showed a heterogenous PSMA index labelling between TMA cores. This particular issue was resolved by performing a cumulative assessment of the tumor area in the analyzed cores and choosing the predominant pattern of expression or, when not possible, eliminating the cases from the cohort.

Therefore, there may be a slight underrepresentation of positive cases due to heterogeneity. Heitkötter et al. compared TMA results with whole slides in 37 tumors from all three categories of biological potential, yielding a discrepancy rate of only ~ 5% [24]. It should be noted that when PSMA immunohistochemistry is performed on biopsies in daily practice, a similar sampling error may occur as when evaluating tissue cores in a tissue microarray.

## Conclusion

Taken together, the current study shows that a considerable subset of soft tissue and bone sarcomas belong to the strong PSMA expression group. The evaluation of PSMA expression through immunohistochemistry can be an accurate and accessible tool in identifying sarcoma patients for PSMA-targeting PET/CT scans and in whom the benefit of PSMA-targeted radioligand therapy may be explored.

## Supplementary Information

Below is the link to the electronic supplementary material.

Supplementary file 1 (PDF 366 KB)
