# Supplementary figures and images for: Neovascular prostate specific membrane antigen (PSMA) expression in bone and soft tissue sarcoma: a systematic analysis
Source: Virchows Arch. 2025 Apr 9;487(6):1299–309. doi: 10.1007/s00428-025-04086-6 (PMC12748178; doi:10.1007/s00428-025-04086-6)

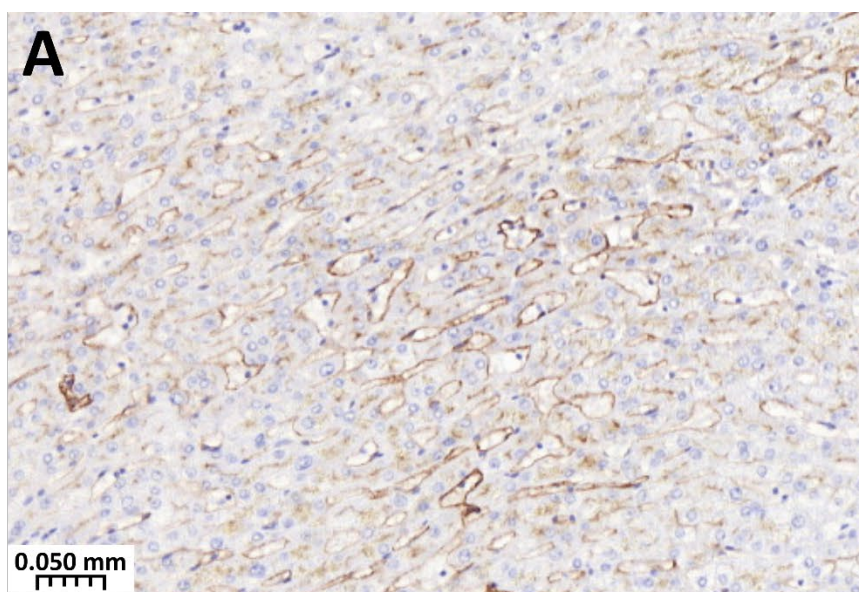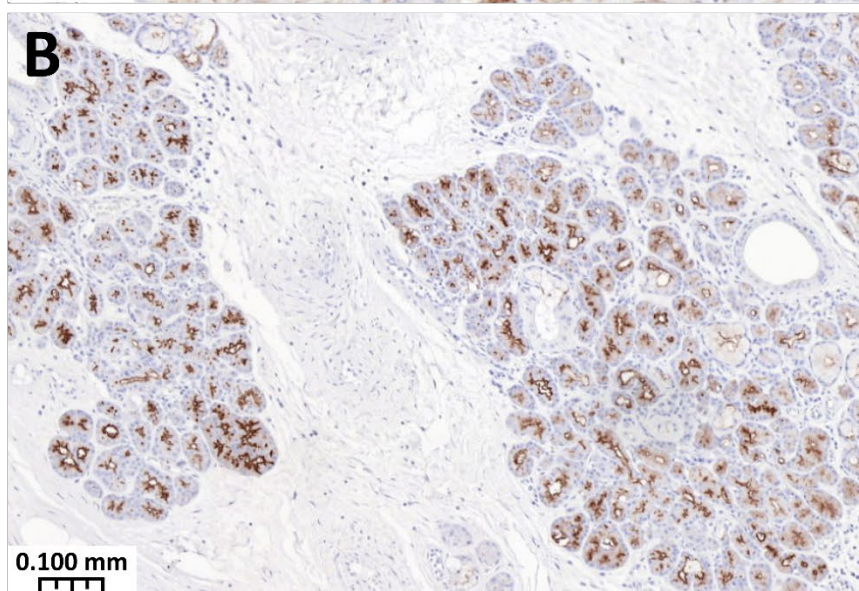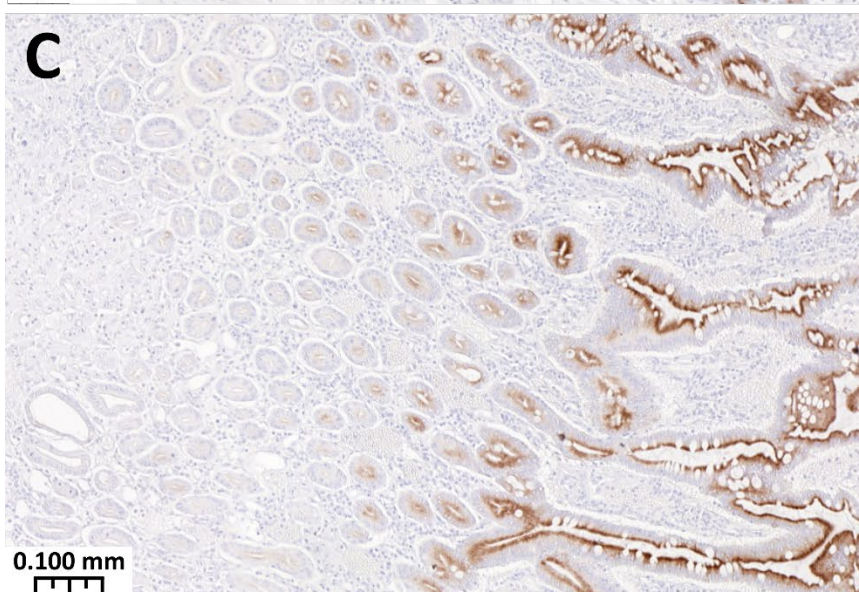

**Supp Figure 1:** PSMA expression in **A:** Liver vessels, **B:** sweat glands, and **C:** small intestine.

Supplement: Supplementary file 1 — (PDF 366 KB) [file 428_2025_4086_MOESM1_ESM.pdf]
